# Malnutrition among under-five children in Democratic Republic of the Congo: A plague of the health system

**DOI:** 10.1016/j.amsu.2022.104260

**Published:** 2022-08-24

**Authors:** Aymar Akilimali, Styves Banga, Malik Olatunde Oduoye, Chrispin Biamba, Ami Munyangi, Elysée Byiringiro, Balagizi Fabien, MUBAGWA Guy Laroche, Wanny Masirika

**Affiliations:** Faculty of Medicine, Official University of Bukavu, Bukavu, D.R, Congo; Department of Research, Research Circle, Bukavu, D.R, Congo; Department of Research, Oli Health Magazine Organization, Kigali, Rwanda; Faculty of Medicine, Official University of Bukavu, Bukavu, D.R, Congo; Department of Research, Research Circle, Bukavu, D.R, Congo; College of Medical Science, Ahmadu Bello University, Zaria, Nigeria; Department of Research, Research Circle, Bukavu, D.R, Congo; Faculty of Medicine, University of Goma, Goma, D.R, Congo; Department of Research, Research Circle, Bukavu, D.R, Congo; Faculty of Medicine, Catholic University of Bukavu, Bukavu, D.R, Congo; Faculty of Medicine, University of Rwanda, Kigali, Rwanda; Department of Research, Research Circle, Bukavu, D.R, Congo; Faculty of Medicine, Evangelic University in Africa, Bukavu, D.R, Congo; Department of Research, Research Circle, Bukavu, D.R, Congo; Faculty of Economy, Official University of Bukavu, Bukavu, D.R, Congo; Department of Research, Research Circle, Bukavu, D.R, Congo; Faculty of Economy, Official University of Bukavu, Bukavu, D.R, Congo

**Keywords:** Child, Democratic republic of the Congo, Malnutrition, Malnutrition complication, Nutritional status

## Abstract

Malnutrition in children has been recognized as a major public health problem in the world and in particular in the Democratic Republic of Congo, which directly contributes to the increase in mortality and morbidity in this already fragile population. The Democratic Republic of Congo is a country plagued by repeated conflicts between different local armed groups and instability in the management of political affairs. There is a high prevalence of malnutrition in the eastern provinces of the country. Although it is a major public health problem, the inadequacy of the system in question plays an important role in the inequality of access to health care and therefore in the lack of growth monitoring of very young children under 5 years old. The rate of malnourished children has been observed in the country. Malnutrition in these children causes several alterations such as delayed physical and motor growth, a decrease in immune defenses which considerably increases the mortality rate and a decrease in cognitive and learning abilities. Malnutrition remains a serious public health problem in low-income countries and is reflected in various statistics from the World Health Organization. Children are more at risk of being at nutritional risk or suffering from malnutrition. This narrative review summarizes current data on the prevalence and determinants of malnutrition in children, including the difficulty of providing children with a consistent food intake due to mass displacement from conflict areas, the inability of nutritional centers to care of patients admitted for malnutrition due to lack of equipment and the many epidemics the country has had to deal with which have severely handicapped the already unstable health system.

## Highlights

• Malnutrition is a plague for the health system of the Democratic Republic of Congo, of which children fewer than 5 are victims.
• Malnutrition is inevitable by ensuring good food security in young children, which reduces the death rate.
• The Congolese government must mobilize all its efforts in the fight against malnutrition among young children in recent days.

## 1 Introduction

Malnutrition is a plague for the health system of the Democratic Republic of Congo (DR Congo). However, it contributes to about 45% of deaths of children fewer than 5 years old [1]. Poverty increases the risk of malnutrition, especially among children who are more vulnerable, malnutrition also increases health care costs and retards economic growth [2]. The increase in the child malnutrition rate in DR Congo is also explained by food insecurity in households [1,3], but also with this pandemic of the Coronavirus disease 2019 (COVID-19), which has destabilized the health systems of many countries, engendering a nutritional crisis which makes the situation increasingly difficult [1].

This study aims to analyze the epidemiological characteristics and factors that aggravate malnutrition in children.

## 2 Epidemiology of malnutrition in DR Congo

Malnutrition remains a serious public health problem worldwide and particularly in low- and middle-income countries. In 2018, the latest Global Nutrition Report shows a prevalence of approximately 50.3 million (7.5%) children suffering from malnutrition and an estimated 5.8 million deaths occur each year among children under 5 years of which 45% are attributed to malnutrition [8].

Climate change as well as natural disasters (volcano) is some of the aggravating factors of malnutrition [4]. The DR Congo, being a large country and the most affected by numerous epidemics of diseases including COVID-19, meningitis, bubonic fever, Ebola etc., which destabilizes its health system [5]. DR Congo has experienced some of the worst humanitarian crises in the world with around 3 million children living on the move in sites where living conditions are even harsher. A sufficient number of children under five (one hundred thousand) and pregnant and breastfeeding women suffer from acute malnutrition. Estimates show that over 200,000 children are severely malnourished and in need have urgent care [6]. Between September 2021 and March 2022, there was a period of peak malnutrition, many health zones in the DR Congo were in a critical nutritional situation. Deterioration occurred in the month of April 2022 because of adequate measures to mitigate the factors aggravating malnutrition which were not taken seriously [7].

## 3 Causes and consequences of malnutrition in DR Congo

Limited disease treatment, monitoring and screening centers, armed conflicts, depletion of resources and lack of health equipment, poor infrastructure are various factors that deteriorate and weaken the health system in DR Congo [5].

The economic, social, medical and developmental consequences of the global burden of malnutrition are severe and persistent for individuals and their families, communities and individuals. According to the World Health Organization (WHO), malnutrition is a causative factor in almost half of the 10.4 million deaths of children under five [9]. Studies show that there are multiple and, sometimes, overlapping consequences. It is difficult to separate one consequence from another [1]. Failure to grow commonly referred to as growth faltering or stunting and wasting, is another consequence of malnutrition. Children who suffer from protein-energy malnutrition are shorter and weigh less, compared with others in an age-gender-specific category [6]. Malnutrition also affects the function and recovery of every organ system, such as muscle function, cardiopulmonary function, and gastrointestinal function, immune and wound healing, and psychosocial effects [9]. These consequences are either short-term or long-term consequences.

## 4 Efforts to overcome malnutrition in the DR Congo

Appropriate strategies to reduce the different forms of malnutrition among children, especially those under 5, should be put in place by the Congolese government as well as the WHO and its partners to improve parents' knowledge of nutritional balance and food composition. Child nutrition programs as well as the food security program must be developed by the government of the DR Congo in order to preserve and strengthen the health of children affected by malnutrition. Currently, mining resources and efforts invested in the mining industry can be important in alleviating the malnutrition crisis in DR Congo if they are diverted to building a more structured health and food system [2].

In addition, the government should improve and strengthen health systems by putting in place the qualified personnel and materials necessary for better care of malnourished children. The Congolese government should also fight to put an end to the armed conflicts that reign in the country from day to day and which make certain regions uninhabited when these regions should be accessible and important in the agricultural sector, which is an important factor in food.

The Congolese government must improve the standard of living of the population by creating jobs, an action that will allow many people to find the means to safely support the balanced diet of their children. Malnutrition is a scourge that currently affects underdeveloped countries and some developing countries in the world. The WHO proposes a plan for a healthy and balanced diet in young children and specially to promote breastfeeding in infants to put an end to this scourge which affects certain corners of the world.

## 5 Recommendations and conclusion

Despite the difficulties caused by the COVID-19 pandemic, the United Nations and its members should mobilize monetary funds for vital assistance to children and families most vulnerable to malnutrition in regions in difficulty but also for the population of the DR Congo, a population living in armed conflicts. The increase in food insecurity and malnutrition is explained by the conflicts and insecurity that reign throughout the DR Congo as well as the limited access to essential nutrition and health services as well as than water, sanitation and hygiene.

We are proposing to the Congolese government to bring its health system into line with nutritional needs and ensure essential and effective nutritional coverage, to strengthen trade policies while improving nutrition and to strengthen and promote everywhere governance and accountability in the field of nutrition. The Congolese government and its partners in the economy and in agriculture must develop agricultural systems and deploy mechanisms to ensure food security for its entire population and for the whole country. This action will be a good contribution to the fight against malnutrition in the Congolese population and young children will benefit from food security. We also recommend an appeal to humanitarian actors throughout the DR Congo to strengthen access and assistance in terms of nutrition, health and water and sanitation services for malnourished children and to their vulnerable families as well as creating sustainable food systems that promote healthy diets, social protection and nutrition awareness at the expense of all.

Malnutrition in children remains a major and serious problem in the health system of the DR Congo, a system destabilized by the various disease epidemics and the COVID-19 pandemic. Malnutrition is preventable but also treatable if you have good pediatric healthcare services. It can be prevented while ensuring good food security in young children to avoid the cases of death that this disease causes.

## Sources of funding

This research did not receive any specific grant from funding a commercial, or not for profit sectors.

## Ethical Approval

Not applicable.

## Consent

Not applicable.

## Author contribution

Conception: Aymar AKILIMALI, Design: Styves BANGA, Administrative support: Aymar AKILIMALI, Literature search: Aymar AKILIMALI and Ami MUNYANGI, Manuscript preparation: Aymar AKILIMALI, Malik OLATUNDE ODUOYE and Elysée BYIRINGIRO, Manuscript editing: Aymar AKILIMALI, MUBAGWA Guy Laroche and Styves BANGA, Manuscript review: Aymar AKILIMALI, Wanny MASIRIKA, Chrispin BIAMBA and BALAGIZI Fabien, Final approval of manuscript: All Authors.

## Registration of research studies

1. Name of the registry: not applicable.
2. Unique Identifying number or registration ID: not applicable.
3. Hyperlink to your specific registration (must be publicly accessible and will be checked): not applicable.

## Guarantor

Aymar AKILIMALI is the guarantor of this study and accept full responsibility for the work and the conduct of the study, has access to the data and controlled the decision to publish.

## Declaration of competing interest

The authors declare that they have no conflicts of interest.

## Abbreviations

COVID-19: Coronavirus disease 2019, DR Congo: Democratic Republic of the Congo, WHO: World Health Organization\
